# Consequences of larval competition and exposure to permethrin for the development of the rodent malaria *Plasmodium berghei* in the mosquito *Anopheles gambiae*

**DOI:** 10.1186/s13071-020-3983-9

**Published:** 2020-02-27

**Authors:** Gaël Hauser, Kevin Thiévent, Jacob C. Koella

**Affiliations:** Institute of Biology, University of Neuchâtel, Rue Emile-Argand 11, 2000 Neuchâtel, Switzerland

**Keywords:** Malaria, *Plasmodium berghei*, *Anopheles gambiae*, Pyrethroids, Sublethal effects

## Abstract

**Background:**

Mosquitoes and other vectors are often exposed to sublethal doses of insecticides. Larvae can be exposed to the run-off of agricultural use, and adults can be irritated by insecticides used against them and move away before they have picked up a lethal dose. This sublethal exposure may affect the success of control of insect-borne diseases, for it may affect the competence of insects to transmit parasites, in particular if the insects are undernourished.

**Methods:**

We assessed how exposure of larvae and adults to a sublethal dose of permethrin (a pyrethroid) and how larval competition for food affect several aspects of the vector competence of the mosquito *Anopheles gambiae* for the malaria parasite *Plasmodium berghei.* We infected mosquitoes with *P. berghei* and measured the longevity and the prevalence and intensity of infection to test for an effect of our treatments.

**Results:**

Our general result was that the exposure to the insecticide helped mosquitoes deal with infection by malaria. Exposure of either larvae or adults decreased the likelihood that mosquitoes were infected by about 20%, but did not effect the parasite load. Exposure also increased the lifespan of infected mosquitoes, but only if they had been reared in competition. Larval competition had no effect on the prevalence of infection, but increased parasite load. These effects may be a consequence of the machinery governing oxidative stress, which underlies the responses of mosquitoes to insecticides, to food stress and to parasites.

**Conclusions:**

We conclude that insecticide residues are likely to affect the ability of mosquitoes to carry and transmit pathogens such as malaria, irrespective of the stage at which they are exposed to the insecticide. Our results stress the need for further studies to consider sublethal doses in the context of vector ecology and vector-borne disease epidemiology.
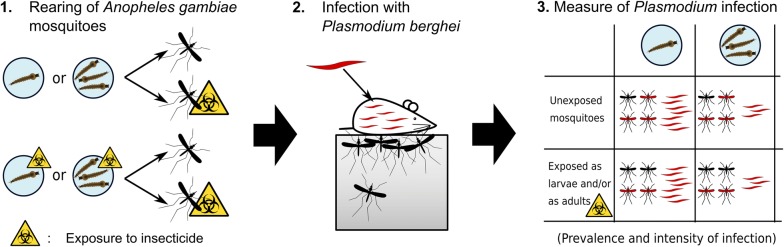

## Background

Mosquitoes and other vectors of infectious diseases are often exposed to sublethal doses of insecticides. On the one hand, when mosquitoes touch the insecticides that are meant to kill them, they can be irritated and fly away before the contact is long enough to do so [1]. On the other hand, the insecticides that are used in agriculture can runoff and end up at sublethal concentrations in surface or ground water, where they can affect many animals, including aquatic arthropods such as mosquito larvae [2].

Such sublethal concentrations can increase oxidative stress [3, 4], modulate the immune responses [5], and increase the activity of detoxifying enzymes [6–8] and the metabolic cost associated with detoxification. They can also affect the behavior of mosquitoes [9–12], in particular if the insecticide is neurotoxic.

Sublethal concentrations of insecticides also affect the development of malaria and other parasites in mosquitoes. Thus, exposing adult mosquitoes to a low concentration of pyrethroid impedes the development of malaria parasites [13–15], and the effects of exposing larvae can carry over to adults to influence their vector competence (that is, their ability to acquire, maintain and transmit parasites [16]) for arboviruses [17, 18] and malaria [19, 20].

Such carry-over effects on the vector competence of mosquitoes are not limited to insecticides. In particular, nutritional stress during the larval stage impacts the susceptibility of *Aedes aegypti* mosquitoes to arboviruses [21–23], and the competence of *Anopheles* mosquitoes for *Plasmodium* [19, 24, 25] or for filarial worms [26]. In addition, as larval nutrition is also an important determinant of the tolerance of mosquitoes to insecticides [27, 28], the two stressors may interact to affect the competence of mosquitoes for the transmission of parasites [19].

Since these environmental factors, e.g. food limitation for larvae and exposure of insecticides to larvae and adults, may often co-occur in nature, we investigated how their combination would affect the development of malaria in adult mosquitoes, using the mosquito *Anopheles gambiae* (*s.s.*), a pyrethroid insecticide (permethrin), and the rodent malaria parasite *Plasmodium berghei*. Although this host-parasite association is not found in nature, it is often used as a model system to study the development of *Plasmodium* parasites in mosquitoes [29]. In contrast to other studies on the impact of larval exposure, which used high concentrations that kill many larvae or exposed larvae only briefly (e.g. [19, 30–33]), we used a sublethal concentration throughout the larval period.

## Methods

We used the insecticide-sensitive Kisumu strain of *Anopheles gambiae* (*s.s.*) [34] and the ANKA strain of the rodent malaria *Plasmodium berghei* (modified to express green fluorescence protein (GFP), obtained from the laboratory of Dr Heussler at the University of Bern, to assess the consequences of larval competition and of larval and adult exposure to a sublethal concentration of permethrin on the longevity of mosquitoes after infection and on the development of the parasite inside the mosquito. The experiment was run in two blocks with identical methods, and was run in an insectary maintained at 26.5 ± 0.5 °C and 70 ± 5% humidity, with a 12:12 light to dark photoperiod.

### Determination of sublethal dose of permethrin

To find a sublethal concentration of insecticide which is nevertheless relevant in nature, we evaluated the effects of three concentrations (0.1, 0.15 and 0.2 µg/l), which are close to the median concentration of permethrin found in surface waters of agricultural areas (0.04 µg/l (25th percentile: 0.01 µg/l, 75th percentile: 0.31 µg/l)) [35]. We created these concentrations by dissolving solid permethrin (Sigma-Aldrich Inc., St. Louis, Missouri) in pure ethanol to obtain a 1 µg/ml stock solution, and then diluting the stock solution in the adequate volume of deionized water. Fourty freshly hatched larvae per concentration were placed individually into glass Petri dishes (4 cm diameter × 1.2 cm height) containing 4 ml of solution, and fed according to the regime given below. To control for the effect of the ethanol, we also tested a solution of 0.015% volume per volume (v/v) (118 µg/l) ethanol. We recorded survival as the proportion of adults emerging at each concentration. The highest concentration of permethrin giving no significant mortality (tested against the mortality of control using a 2-sample test for equality of proportions (*prop.test* in R)) was 0.15 µg/l, so we used this concentration as our sublethal dose, keeping the concentration of ethanol (for unexposed and exposed larvae) at 0.015% v/v (118 µg/l).

### Mosquito rearing and permethrin exposure

Freshly hatched larvae (0–3 hours-old) were reared in glass Petri dishes containing 4 ml of 0.015% v/v (118 µg/l) ethanol in deionized water with 0.15 µg/l permethrin or no permethrin. The larvae were reared individually or in groups of three. They were provided daily with Tetramin Baby® fish food according to their age: 0.04, 0.06, 0.08, 0.16, 0.32 and 0.6 mg/Petri dish for ages 0, 1, 2, 3, 4 and 5 days or older, respectively [28]. Note that this gives strong competition for food in the Petri dishes containing three larvae. Upon pupation, mosquitoes were moved individually into 50 ml Falcon™ tubes. We discarded the pupae of the competition treatment, if any of their competitors within a Petri dish had died. If more than one female emerged from the same Petri dish, we randomly selected one of them with a random number generator and discarded the others to guarantee independence of data. After emergence, males were discarded, and females were moved to cages and given continuous access to a 6% sucrose solution. Fourty hours before infection (so when females were between 1 and 5 days-old), half of the females were moved in groups of 25 to WHO insecticide-testing tubes and exposed for 2 minutes to permethrin 0.75% treated papers, and the other half were exposed to WHO control papers according to the WHO exposure protocol [36]. Exposed mosquitoes were moved back to cages according to their treatment. One mosquito, which had been reared individually and not exposed to permethrin at it larval stage, died within 24 hours of its exposure to permethrin. No mosquito died after the sham exposure.

### Infection with *Plasmodium berghei*

Two infected mice were available for each block. To control for differences among mice, we split the mosquitoes of each treatment into two cages, one for each mouse. Each mouse was used to feed one cage per treatment (so a total of 8 cages); the order of the treatments was opposite for the two mice. The mice were anesthetized with an intra-peritoneal injection (8.5 ml/kg) of a solution of Xylazine Xylasol® (20 mg/ml), Ketamine Ketasol (100 mg/ml) and PBS [37], and placed onto each cage for 10 minutes.

Mosquitoes were infected 40 hours after they had been exposed to permethrin or the control paper (so 3 to 7 days after emergence). Twenty-four hours before the infection, the temperature was gradually lowered to 19 °C, and it was maintained at this level for the rest of the experiment to allow the development of *P. berghei* [38]. The parasite takes about 10 days to develop into oocysts, and another 10 days to invade the salivary glands as infectious sporozoites [38, 39]. As we were interested in the infectious stage, mosquitoes were kept for 22 days. During this time, mortality was recorded daily and dead mosquitoes were frozen at − 20 °C.

22 days after infection, remaining mosquitoes were killed by freezing and kept at − 20 °C before being further assessed for their infection status and parasite load.

### Measuring wing lengths

The lengths of the left wings of the mosquitoes were measured from the axillary incision to the tip of the wing [40] with the software ImageJ [41].

### Measuring infection

To determine the infection of the mosquitoes we dissected them in 0.15 M NaCl solution. We counted the oocysts under a fluorescence microscope, and transferred the salivary glands to 1.5 ml Eppendorf tubes containing 10 μl of Triton X-100 0.05 %, and kept them at − 80 °C until the number of sporozoites was assayed with a real-time PCR. Note that we counted the oocysts because of the possibility that some parasites had not yet developed into sporozoites but would remain visible as oocysts. Therefore, sporozoite-negative mosquitoes were only classified as uninfected if they were also oocyst-negative.

#### DNA extraction

The extraction protocol was modified from Rider et al [42]. Salivary glands were crushed in 125 µl of DNAzol® (MRC Inc. Cincinnati, Ohio) using micro pestles. The resulting homogenate was incubated at 55 °C for 20 min, and centrifuged at 20,000×*g* for 10 min. 100 µl of the supernatant were transferred to a new tube, which had previously been filled with 1.5 µl of Polyacryl carrier (MRC Inc. Cincinnati, Ohio) to increase DNA recovery, and 100 µl of ethanol 100% were added to the tube to induce DNA precipitation. The tubes were centrifuged at 15,000×*g* for 8 min, and the supernatant was discarded. The resulting DNA pellet was washed with 600 µl of ethanol 75% and the tubes were centrifugated at 15,000×*g* for 5 min. Ethanol was discarded, and DNA pellets were dried using a speedvac at 45 °C for about 20 min. Dry DNA was eluted in 20 µl of milli-Q water and kept at −80 °C.

#### Real-time PCR

Real-time PCR was performed with a LightCycler 96 ® system (Roche, Switzerland). A master mix was prepared with 4 µl of HOT FIREPol EvaGreen qPCR Mix Plus (ROX) (Solis Biodyne, Estonia), 0.8 µl of each of the primers (400 nM final concentration), 3 µl of extracted DNA, and 11.4 µl of Nuclease-Free Water (Qiagen, Germany). We used primers that amplify 111 base pairs in block 4 of the merozoite surface protein-1 gene (MSP-1) of *P. berghei* ANKA and NK65 strains, as described in [42]. Real-time amplifications steps were set to 95 °C for 15 min (initial denaturation, required for EvaGreen qPCR Mix), followed by 50 cycles of 3-step amplification implying (i) 95 °C for 30 s (denaturation), (ii) 53 °C for 45 s (annealing), and (iii) 72 °C for 30 s (extension). Samples were tested in duplicates in 96-well plates, along with standard dilutions (from 10^8^ to 10^1^ gene sequences per µl, see below) in duplicates, blanks, and negative control (extracted DNA from uninfected mosquitoes from our lab colony). The use of EvaGreen dye qPCR mix was already tested for *Plasmodium* quantification in [43] and showed satisfying results. Samples were assayed in duplicates, and showed a repeatability of 0.976.

#### MSP-1 standards

*Escherichia coli* carrying a plasmid with the 111 base pair sequence obtained from a PCR amplification were cultured in LB medium. Plasmid DNA was isolated using Wizard® Plus SV Minipreps DNA Purification System (Promega Corp., Wisconsin). DNA concentration in the purified product was measured using Nanodrop (Thermo Fisher Scientific Inc., Massachusetts, USA), and the number of gene sequences was estimated from the known molecular weight of our target sequence. Serial dilutions were finally made to obtain standards of concentrations ranging from 10^8^ to 10^1^ sequences per µl.

### Statistical analyses

All analyses were done with the software R (version 3.4.4) [44]. The significance of the effects was assessed with the ANOVA function of the *car* library [45], using a type III ANOVA if an interaction was significant, and a type II ANOVA otherwise. Non-significant interactions were dropped from the final models. In cases of significant interactions contrast analyses were done between the factors of interest using *emmeans* (computing Estimated Marginal Means (EMM)) and *pairs* functions of the *emmeans* library in R, with p-values being adjusted using the *mvt* method. Analysed datasets are published as supplementary information files: larval development (Additional file 1: Table S1), survival and infection parameters (Additional file 2: Table S2), and permethrin lethality trial (determination of the dose of permethrin, Additional file 3: Table S3).

#### Larval development

Mortality during development was analyzed with a Generalized Linear Model (GLM) with a quasibinomial distribution of errors. The response variable was the proportion of dead mosquitoes per Petri dish (so, 0 or 1 if larvae were reared individually, and 0, 0.33, 0.67 or 1 if larvae were reared in groups of three. Explanatory variables were competition status and larval exposure to permethrin. Block was included as a co-factor.

We used a Cox’s proportional hazard model from the *survival* library in R [46] to analyze the effect of competition and larval exposure to permethrin on development time (so, the number of days from hatching to pupation). For the competition treatment, the average development time for each Petri dish was considered as the response variable. Petri dishes in which one or more larva died were censored on the day of the first death recorded. Block was set as a co-factor.

#### Wing length

We used a Linear Model (LM) to test for any difference of wing length among the larval treatments. The response variable was wing length, and explanatory variables were larval competition status and larval exposure to permethrin. Block was included as a co-factor. As wing length is usually strongly linked to competition, we did not consider it as a co-factor in other analyses to avoid a false interpretation of the link between competition and the other traits of interest.

#### Longevity

Post-infection longevity was analyzed with a Cox’s proportional hazard model from the *survival* library [46] in R. Day zero was set as the day of infection. 22 days after infection, living mosquitoes were censored. Explanatory variables were larval competition status, larval exposure to permethrin, and adult exposure to permethrin. The blocks and the mice nested within the blocks were included as co-factors. The proportional hazard ratio assumption was tested using the function *cox.zph* from the *survival* library.

#### Plasmodium infection

To analyze the effect of larval competition and larval and adult exposure to permethrin on *Plasmodium* infection, we first assessed the prevalence of infection in the different treatments and then assessed the oocyst load and the sporozoite load in infected individuals.

A mosquito was considered as infected if either oocysts or sporozoites were found, and it was considered infectious if sporozoites were found. We used a Generalized Linear Model (GLM) with binomial error distribution to analyze both the prevalence of infection and infectiousness among our different treatments: larval competition, larval exposure to permethrin, and adult exposure to permethrin. The blocks and the mice nested within the blocks were included as co-factors.

The effects of the treatments on oocyst and sporozoite loads were analyzed with a Linear Model (LM). The same variables were included as for the analysis of infection. The parasite loads were log-transformed to reach normality.

We also tested the proportion of mosquitoes that were infected with oocysts but not (yet) sporozoites, which could be a measure of the rate of parasite development. That proportion was tested with a binomial GLM that included competition, larval exposure and adult exposure as explanatory factors, and the block and the mouse nested within the blocks were included as co-factors.

## Results

### Larval development

Larval mortality ranged from 2.8% to 12.3%. Exposure to permethrin (*χ*^2^ = 13.8, *df* = 1, *P* < 0.001) and competition (*χ*^2^ = 5.7, *df* = 1, *P* = 0.017) increased mortality, and there was a significant interaction between the two factors (*χ*^2^ = 4.9, *df* = 1, *P* = 0.027). Permethrin increased mortality from 2.8% (95% CI: 1.3–6.2%) to 12.3% (95% CI: 8.5–17.6 %) (*z* = 3.3, *P* = 0.001) in larvae reared by groups of three, but had a small effect if larvae had been reared individually (from 7.5% (95% CI: 5.2–10.7%) to 11.1% (95% CI: 8.3–14.7%); contrast analysis: *z* = 1.7, *P* = 0.097).

Almost all larvae (96%) pupated between 7 and 12 days after hatching. The 67 of 1682 larvae that had not pupated by day 12 were removed from the experiment. Competition increased development time from 7.7 ± 0.05 to 9.6 ± 0.07 (mean ± 95% CI) (*χ*^2^ = 584.9, *df* = 1, *P* < 0.001). There was no main effect of permethrin (*χ*^2^ = 0.8, *df* = 1, *P* = 0.37), but there was an interaction between the two factors (*χ*^2^ = 50.7, *df* = 1, *P* < 0.001). Permethrin exposure increased age at pupation in larvae reared individually from 7.41 ± 0.06 to 8.03 ± 0.08 days (mean ± 95% CI) (contrast analysis: *z* = 12.9, *P* < 0.001), but had no effect on larvae reared by groups of three (from 9.55 ± 0.09 to 9.65 ± 0.10 days; z = 0.9, *P* = 0.37).

### Wing length

Larval competition for food decreased wing length from 3.16 ± 0.02 mm (for individual reared larvae) to 2.85 ± 0.02 mm (*F*_(1, 425)_ = 378.98, *P* < 0.001), and larval exposure to permethrin marginally increased wing length from 3.01 ± 0.03 mm to 3.03 ± 0.02 mm (*F*_(1, 425)_ = 4.99, *P* = 0.026). There was a significant interaction between the two factors (*F*_(1, 425)_ = 4.90, *P* = 0.027); permethrin-exposed mosquitoes were slightly larger (2.88 ± 0.03 mm) than unexposed ones (2.84 ± 0.02 mm) if larvae had been reared in groups of three (from contrast analysis: *t* = − 2.23, *df* = 420, *P* = 0.026), but exposure had no effect if larvae had been reared individually (3.15 ± 0.02 mm if exposed and 3.17 ± 0.02 mm if unexposed; contrast analysis: *t* = 0.85, *df* = 420, *P* = 0.39).

### Post-infection longevity

**Fig. 1:**
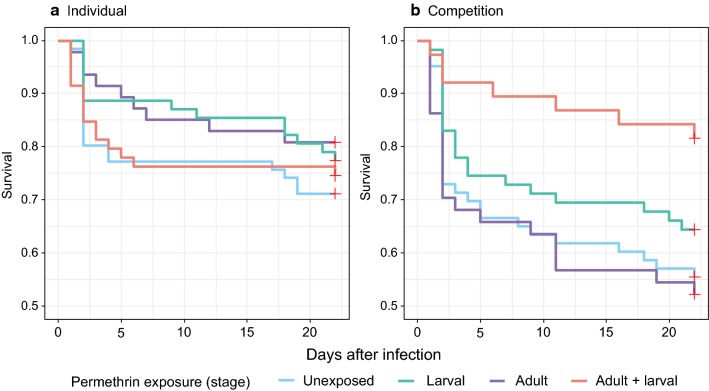
**a** Post-infection longevity according to permethrin exposure for mosquitoes that had been reared individually. Sample sizes: unexposed: *n* = 66; larval: *n* = 62; adult: *n* = 47; adult and larval: *n* = 59. **b** Post-infection longevity according to permethrin exposure for mosquitoes that had been reared in competition. Sample sizes: unexposed: *n* = 63; larval: *n* = 59; adult: *n* = 44; adult and larval: *n* = 38. Scale of the y-axis was fixed between 0.5 and 1 to improve visibility

In total, 438 mosquito females fed on the infected mice and were kept for the rest of the experiment. Among them, 234 females were reared individually (66 unexposed, 62 exposed as larvae, 47 as adults, and 59 as larvae and adults), and 204 females were reared in competition (63 unexposed, 59 as larvae, 44 as adults, and 38 as larvae and adults). Survival 22 days after the blood meal ranged from 52.3% (95% CI: 37.9–66.2%) for mosquitoes reared in competition and exposed to permethrin only as adults to 81.6% (95% CI: 66.6–90.8%) for mosquitoes reared in competition and exposed both as larvae and adults. Competition slightly decreased longevity (*χ*^2^ = 3.7, *df* = 1, *P* = 0.054). Neither larval (*χ*^2^ = 1.6, *df* = 1, *P* = 0.21) nor adult (*χ*^2^ = 0.4, *df* = 1, *P* = 0.55) exposure to permethrin had a main effect on longevity, but there was an interaction between competition and the two exposures (*χ*^2^ = 4.3, *df* = 1, *P* = 0.037). Contrast analysis showed that when mosquitoes were reared individually, longevity did not differ between the treatments (all *P* > 0.68, Fig. 1a). However, in mosquitoes reared in groups of three, those that were exposed to permethrin as larvae and as adults lived longer than those that were either exposed only as adults (*z* = 2.8, *P* = 0.025) or those that were not exposed to permethrin (*z* = 2.48, *P* = 0.059) (Fig. 1b). Other contrasts were not significant (*P* > 0.3).

### *Plasmodium* infection

The output of the models for malaria infection (infection prevalence, infectiousness, sporozoite load and oocyst load) are presented in Table 1. Reported results were consistent among the two experimental blocks.

**Table 1:** Results of the models for the overall infection prevalence (proportion of mosquitoes infected with either sporozoites, oocysts, or both), infectiousness (proportion of mosquitoes infected with sporozoites), sporozoite load, and oocyst load

Treatment	Infection prevalence		Infectiousness		Sporozoite load		Oocyst load	
	*χ*^2^	*P*	*χ*^2^	P	*χ*^2^	*P*	*χ*^2^	*P*
Competition	0.43	0.51	0.13	0.71	3.79	0.053	12.64	**<** **0.001**
Larval exposure	10.14	**0.001**	11.94	**<** **0.001**	0.07	0.78	4.53	**0.035**
Adult exposure	8.24	**0.004**	5.64	**0.018**	1.68_1_	0.20	4.22	**0.042**
Competition: larval exposure	0.06	0.80	0.01	0.91	0.06	0.81	8.21	**0.005**
Competition:adult exposure	0.17	0.68	0.01	0.94	0.38	0.54	1.05	0.31
Larval exposure: adult exposure	7.87	**0.005**	6.38	**0.011**	0.18	0.67	0.46	0.50
Competition:larval exposure: adult exposure	0.01	0.93	0.15	0.70	1.23	0.27	1.68	0.20

*Notes*: The Chi-square values and the *P*-values are given. All degrees of freedom are 1. *P*-values in bold indicate statistical significance

#### Overall infection

**Fig. 2:**
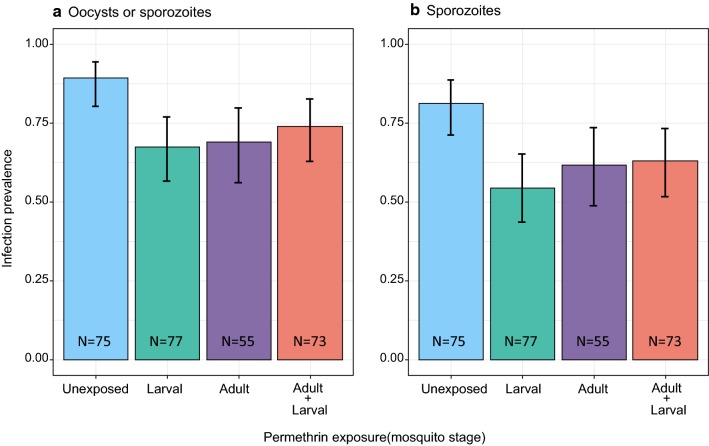
**a** The prevalence of infection of the mosquitoes 22 days after the infectious blood meal. Mosquitoes were considered as being infected if they harbored either oocysts, sporozoites or both. Colors indicate mosquitoes’ exposure to permethrin. **b** Mosquitoes’ infectiousness, i.e. the proportion of mosquitoes harboring sporozoites 22 days after the infectious blood meal. Error bars show the 95% confidence intervals

303 mosquitoes were killed and dissected 22 days after infection. We counted the oocysts in all 303, but could recover salivary glands and determine the sporozoite load in only 280. To analyse the overall prevalence of infection (that is, independently of the parasite’s stage), we used only mosquitoes for which both oocyst and sporozoite load could be determined. 75.4 % (95% CI: 69.9–80.0 %) of the mosquitoes were infected. Competition status did not affect the proportion of infected individuals, with 76.7% (95% CI: 69.6–82.5%) in mosquitoes reared individually against 73.5% (58.8–80.7%) in those reared by groups of three (Table 1). The prevalence in unexposed larvae was 89.3% (95% CI: 80.3–94.5%) and was significantly lower if mosquitoes were exposed as larvae (67.5% (95% CI: 56.5–76.9%)), as adults (69.1% (95% CI: 56.0–79.7%)), or as larvae and adults (74.0% (95% CI: 62.9–82.7%)) (Table 1, Fig. 2a).

#### Sporozoites

183 of the 280 salivary glands were infected with sporozoites (65.4 % (95% CI: 59.6–70.7 %)). In unexposed mosquitoes, the prevalence was 81.3% (95% CI: 71.1–88.5%). The prevalence was significantly lower if mosquitoes were exposed at larval stage or adult stage. Indeed, it was 54.5% (95% CI: 43.5–65.2%) if larvae had been exposed, 61.8% (95% CI: 48.6–73.5%) if adults had been exposed and 63.5% (95% CI: 53.5–73.2%) if mosquitoes had been exposed as larvae and adults (Table 1, Fig. 2b). Competition had no effect on the mosquitoes’ infectiousness (64.4% (95% CI: 56.8–71.4%) for individually reared larvae; 66.7% (95% CI: 57.7–74.6%) for mosquitoes reared in groups of three) (Table 1).

**Fig. 3:**
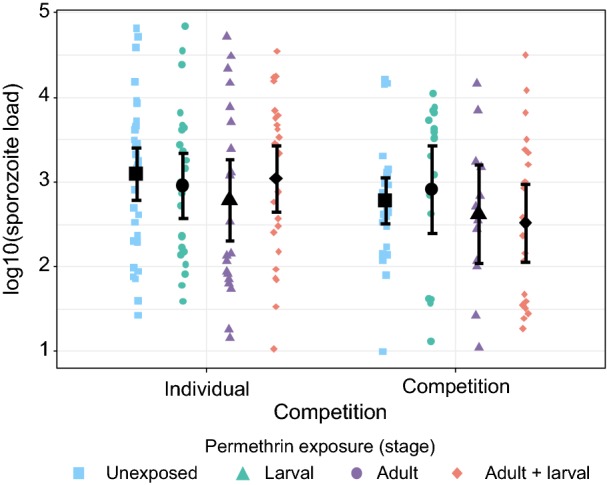
Sporozoite load in mosquito salivary glands. Only mosquitoes harboring sporozoites are shown. All treatments are represented (see figure legend). Error bars show the 95% confidence intervals. Sample sizes: (i) mosquitoes reared individually: unexposed: *n* = 34; larval: *n* = 24; adult: *n* = 22; adult and larval: *n* = 25; (ii) mosquitoes reared in competition: unexposed: *n* = 27; larval: *n* = 18; adult: *n* = 12; adult and larval: *n* = 21

The sporozoite load of sporozoite-positive mosquitoes ranged from 10 to 68,570 (mean ± 95% CI: 4725 ± 1549). Mosquitoes reared in groups of three tended to have fewer sporozoites (2623 ± 1135.8) than individually reared mosquitoes (6286 ± 2543.3) (Table 1, Fig. 3). Permethrin exposure did not affect sporozoite load (Table 1).

#### Oocysts

163 of the 303 mosquitoes harbored oocysts (53.8 % (95% CI: 48.2–59.3 %)). The number of oocysts varied from 1 to 110 (mean ± 95% CI: 17.1 ± 2.62). Mosquitoes reared in groups of three had fewer oocysts (8.4 ± 2.8) than mosquitoes reared individually (15.1 ± 3.7; Table 1). Mosquitoes exposed as adults had fewer oocysts (10.5 ± 3.9) than unexposed ones (14.5 ± 3.5; Table 1). Although overall there was also a lower oocyst load in mosquitoes exposed as larvae (12.0 ± 3.5 *vs* 14.0 ± 4.0; Table 1), larval exposure decreased the oocyst load of mosquitoes only if they had been reared individually (contrast analysis; *t* = 1.9, *df* = 155, *P* = 0.057), but increased it if they had been reared in groups of three (*t* = − 2.1, *df* = 155, *P* = 0.03).

28 out of 280 mosquitoes harbored oocysts but no sporozoites (10.0 % (95% CI: 7.0–14.1%)). This proportion was not affected by any of the treatments (*P* > 0.14) or interaction (*P* > 0.76).

## Discussion

Our general results are that exposure to a low, sublethal concentration of permethrin affected the development and life-history of mosquitoes in ways that impacted the transmission of malaria, and that these effects were influenced by competition for food.

### Mosquito development and adult size

Although we used a concentration of permethrin that we had tested to be sublethal, slightly more exposed than unexposed larvae died, in particular if they had been reared in competition (so, with limited food). Since the exposed mosquitoes that had been reared in competition also developed into slightly larger adults, the insecticide appears to have mainly killed the smallest and weakest larvae. Exposure to permethrin also delayed pupation, but only if mosquitoes were reared individually. This might again reflect that, in competition, the weakest (and thus most slowly developing) larvae were killed by the insecticide.

The effects on the mosquitoes’ development may be a direct consequence of the neurotoxicity of permethrin. They may, however, also be caused indirectly through the costs associated with the mosquitoes’ ways of dealing with (so detoxifying, metabolizing or sequestering) the insecticide [47–49]. If such a cost uses energy as its currency, it should be more apparent in cases of food restriction. Our results were consistent with this idea, as permethrin slightly increased mortality only if larvae had been reared in competition.

### Mosquito longevity post-infection

That, overall, competition decreased the longevity of the mosquitoes corroborates previous work [25] and may be a consequence of the reduced resource availability, leading to small females [50] and limiting mosquitoes’ immune responses (e.g. [5, 51]). Similarly, that permethrin exposure did not affect the longevity of individually reared mosquitoes corroborates studies that found no or negative effect of insecticide on post-infection survival following larvicidal (*Bti*) [19] or pyrethroid exposure [15, 52]. However, in our experiment mosquitoes that were exposed both as larvae and as adults survived longer than unexposed ones, if they had been reared with competition. This suggests that the insecticide alleviate the costs of the infection, perhaps by helping mosquito immunity [5], as suggested by the results on prevalence. In individually reared mosquitoes, however, the high survival rate 22 days after the infection (*c.*80%) may have hidden any beneficial effect of permethrin exposure.

### Infection prevalence and intensity

In contrast to what we found for longevity and to what has been found in other studies [19, 25], larval competition did not influence the prevalence of infection or the proportion of infectious mosquitoes.

However, exposure to permethrin at either larval or adult mosquitoes reduced both the prevalence of infection and the mosquitoes’ infectiousness. These results corroborate several studies that found lower *Plasmodium* infection prevalence after adult mosquitoes were exposed to deltamethrin (a pyrethroid) [14, 15, 52], bendiocarb (a carbamate) or DDT (an organochlorine) [53] (but see [54]). Our findings also complement these results by showing that exposure at larval and adult stages, even at a very low dose, have similar effects on infection prevalence.

The direct effect of pyrethroid insecticides on the development of *Plasmodium* [13, 14] is unlikely to be the only mechanism underlying our results, for larval and adult exposure led to a similar decrease in prevalence. Alternatively, the observed effects may be due to an indirect effect of the insecticide on mosquito physiology and immunity. Insecticides can affect insect immunity in several ways (reviewed in [55]), including effects on the number of hemocytes [56], on the activity of immune enzymes [57, 58], on the melanisation and antibacterial responses [5] and on oxidative stress. Indeed, pyrethroid insecticides are a major source of reactive oxygen species (ROS) in exposed organisms [3, 4, 59], and ROS are a common immune defense against many pathogens [60], including *Plasmodium* [61–63]. In addition, antioxidants are produced in response to pyrethroid exposure [7] and are associated to both pyrethroid tolerance [4, 64, 65] and mosquito’s response to an infectious blood meal [63, 66]. For example, two important cytosolic antioxidants (Cu–Zn SOD2 and SOD3A) have been found to be overexpressed both after an infectious blood meal [66, 67] and in pyrethroid-tolerant *An. gambiae* [4], and are therefore involved in the physiological response of both stressors. Thus, a combination of oxidants (due to exposure to permethrin) and antioxidants (as a response to oxidative stress) may help pyrethroid-exposed mosquitoes to resist a *Plasmodium* infection.

In contrast to what was found with infection prevalence, sporozoite load was decreased by larval competition. As the oocyst load was also reduced in this treatment, the lower sporozoite load is, in all likelihood, not an indication of delayed parasite development. This reduction in parasite load may be explained by the difference in body size, as was the case (at least for oocysts) in a field study in Tanzania [68]. In larger hosts, malaria parasites might have had more energetic resources to develop. It should however be noted that this result does not support the findings of Emami and her team who found correlation between *P. falciparum* load and *A. gambiae* body size [69]. Alternatively, it can also be explained by the higher mortality observed in mosquitoes from competition treatment: heavily infected mosquitoes may not have survived, which led to the observed difference in sporozoite load.

Finally, exposure to permethrin did not affect sporozoite load, despite its impact on infection prevalence and, to a lesser extent, on oocyst load. The global decrease in oocyst load in exposed mosquitoes suggest that the absence of effects on sporozoites is not caused by a delayed parasite development. However, that there was no effect of the insecticide on sporozoite load differs from a study using deltamethrin insecticide [15]. One possible explanation is that the high variability observed in sporozoite load, by decreasing statistical power, affected our chance to detect significant differences between our treatments (Fig. 3). In addition, it should also be noted that *A. gambiae* is not the natural vector of *P. berghei*, and that an infection with the latter often results in much higher parasite load than what is observed with *P. falciparum* for example [70]. Such an overload may also have contributed to hide a possible effect of our treatment on the intensity of infection.

## Conclusions

To conclude, our results show that sublethal exposure to permethrin at larval or adult stages can affect the way mosquitoes respond to a *Plasmodium* infection. We found that exposure to permethrin increased the post-infection longevity of mosquitoes reared in competition, and exposure at both larval and adult stages reduced infection prevalence. To explain these results, we propose oxidative stress and antioxidant activity to be modulated by the insecticide, which in turn helps mosquitoes to limit parasite development. In addition, we showed that larval competition for food, ubiquitous in nature, has direct effects on the outcome of an infection in terms of survival and parasite load, and could modify the way insecticides affect mosquitoes development and survival. Altogether, this suggests that both early life conditions and adult exposure can strongly affect mosquito vector competence.

## Supplementary information

**Additional file 1: Table S1.** Development of mosquito larvae.

**Additional file 2: Table S2.** Longevity and infection parameters of mosquitoes infected with *P. berghei*.

**Additional file 3: Table S3.** Permethrin lethality trial.

## Data Availability

All data generated or analysed during this study are included in this published article and its additional files.
